# In Vivo Analysis of the Biocompatibility and Bone Healing Capacity of a Novel Bone Grafting Material Combined with Hyaluronic Acid

**DOI:** 10.3390/ijms22094818

**Published:** 2021-05-01

**Authors:** Annica Pröhl, Milijana Batinic, Said Alkildani, Michael Hahn, Milena Radenkovic, Stevo Najman, Ole Jung, Mike Barbeck

**Affiliations:** 1 BerlinAnalytix GmbH, 12109 Berlin, Germany; annica.proehl@berlinanalytix.com (A.P.); milijana.batinic@berlinanalytix.com (M.B.); saidkildani@gmail.com (S.A.); 2 Institute of Osteology and Biomechanics, Eppendorf University Hospital, University of Hamburg, 20246 Hamburg, Germany; hahn@uke.de; 3 Department for Cell and Tissue Engineering, Faculty of Medicine, University of Niš, 18108 Niš, Serbia; milena1390nis@gmail.com (M.R.); stevo.najman@gmail.com (S.N.); 4 Department of Biology and Human Genetics, Faculty of Medicine, University of Niš, 18108 Niš, Serbia; 5 Clinic and Policlinic for Dermatology and Venereology, University Medical Center Rostock, 18057 Rostock, Germany; ole.tiberius.jung@gmail.com; 6 Department of Ceramic Materials, Chair of Advanced Ceramic Materials, Institute for Materials Science and Technologies, Technical University Berlin, 10623 Berlin, Germany

**Keywords:** bone regeneration, inflammation, hyaluronic acid, xenogeneic bone graft, immune response, macrophages

## Abstract

The present in vivo study analyses both the inflammatory tissue reactions and the bone healing capacity of a newly developed bone substitute material (BSM) based on xenogeneic bone substitute granules combined with hyaluronate (HY) as a water-binding molecule. The results of the hyaluronate containing bone substitute material (BSM) were compared to a control xenogeneic BSM of the same chemical composition and a sham operation group up to 16 weeks *post implantationem*. A major focus of the study was to analyze the residual hyaluronate and its effects on the material-dependent healing behavior and the inflammatory tissue responses. The study included 63 male Wistar rats using the calvaria implantation model for 2, 8, and 16 weeks *post implantationem*. Established and Good Laboratory Practice (GLP)-conforming histological, histopathological, and histomorphometrical analysis methods were conducted. The results showed that the new hyaluronate containing BSM was gradually integrated within newly formed bone up to the end of the study that ended in a condition of complete bone defect healing. Thereby, no differences to the healing capacity of the control BSM were found. However, the bone formation in both groups was continuously significantly higher compared to the sham operation group. Additionally, no differences in the (inflammatory) tissue response that was analyzed via qualitative and (semi-) quantitative methods were found. Interestingly, no differences were found between the numbers of pro- and anti-inflammatory macrophages between the three study groups over the entire course of the study. No signs of the HY as a water-binding part of the BSM were histologically detectable at any of the study time points, altogether the results of the present study show that HY allows for an optimal material-associated bone tissue healing comparable to the control xenogeneic BSM. The added HY seems to be degraded within a very short time period of less than 2 weeks so that the remaining BSM granules allow for a gradual osteoconductive bone regeneration. Additionally, no differences between the inflammatory tissue reactions in both material groups and the sham operation group were found. Thus, the new hyaluronate containing xenogeneic BSM and also the control BSM have been shown to be fully biocompatible without any differences regarding bone regeneration.

## 1. Introduction

The clinical application of bone substitute materials (BSM) has become an integral part of daily routine in various parts of medicine and dentistry. The gold standard is still autogenous bone won from different intraoral or extraoral donor sites due to its osteoinductive, osteogenic, and osteoconductive properties [1,2,3]. However, its application is associated with different complications such as donor site morbidity, nerve lesions, and in general the need of a second surgical site with a second pain situation and inflammation risks [4,5]. It has been revealed that BSM perform equally to autologous bone transplants, superseding their application in many indications [6,7,8]. In particular, animal derived xenografts are the most widely used BSM due to both their natural chemical composition and the structure that represents the trabecular framework, including the macro- and microporosity as well as the ultrastructure or nanotopography of the hydroxyapatite-based bone matrix [9,10]. A variety of preclinical and clinical studies have already shown that the application of xenogeneic BSMs lead to predictable bone formation due to their excellent osteoconductive properties [11,12,13]. In this context, it has been revealed that xenogeneic BSMs are beneficial for oral and maxillofacial surgery in indications such as sinus augmentation procedures, providing a long-term scaffold for successful subsequent insertion of dental implants [14,15].

Due to the different indications in oral surgery, a broad variety of xenogeneic BSM is available in various delivery forms, ranging from bone blocks over granules to bone pastes [16]. Moldable BSMs have already been shown to be advantageous in indications such as socket preservation, sinus augmentation, and treatment of bone cysts [17,18]. Their viscous properties allow for an optimal filling of irregularly shaped bone defects up to the defect borders [19]. Not least, their viscosity enables an improved handling that helps clinicians to overcome implantation failures such as over- or under-packing of defect sites [19,20]. 

The currently available bone pastes are two-component biomaterials combining synthetic calcium phosphate (CaP)-based BSM granules with a hydrogel composed of polymers such as collagen or hyaluronic acid (HY) due to their high-water binding capacity [19,21]. It is generally assumed that the added extracellular matrix proteins also enable an enhancement of bone healing by triggering molecular processes such as cell adhesion, proliferation, and migration of osteoblast and other related cascades [22,23]. Furthermore, it has been shown that cell types such as endothelial cells as well as the implantation bed vascularization can be increased by such molecules via an increased cell migration or angiogenesis [24]. Thereby, it has revealed that the application of BSM with a paste-like consistency seem to induce a special integration pattern in accordance with the process of Guided Bone Regeneration (GBR), guiding a cell and vessel ingrowth from the periphery towards the implant core [25]. Finally, different study results revealed that an addition of collagen or HY increased osseous healing [26,27,28]. 

In addition, the biomaterial-associated immune response has received increasing attention in recent years, as a primarily anti-inflammatory approach can improve the healing of (bone) tissue defects [29,30]. In this context, it has been reported that even high molecular weight HY (HMWHY) triggered the polarity of macrophages towards an M2-phenotype, which is supposed to contribute to the overall anti-inflammatory alignment of the material-related foreign body reaction and to improve bone tissue regeneration [31]. In contrast, it was presented in another in vivo experiment that the addition of two different amounts of HMWHY, combined with a synthetic biphasic BSM, to an injectable BSM did not affect the inflammatory response, while only improving the material handling properties [32]. In this context, it is known that the unstabilized HY molecule has a relatively short half-life in the body of between 17 hours and 1.5 days [33,34]. Thus, it can be concluded that the HY molecules integrated in injectable BSM are most often degraded within a short time span after implantation into the bony defects before having any effect on the tissue or healing response. Altogether, these results lead to the question of the biological and regenerative activity of HY for bone tissue regeneration. Even in view of the development of a new class of immunoregulating biomaterials such as BSM, this question needs to be clarified.

Thus, the present in vivo study was conducted, analyzing the (inflammatory) tissue reaction in combination with the bone healing capacities of a newly developed bone grafting material based on a well-researched xenogeneic BSM combined with HMWHY. Previously established methods were applied after implantation of the injectable BSM using the calvaria model in Wistar rats [35,36,37].

## 2. Results

### 2.1. Results of the Histological Analysis

**Figure 1:**
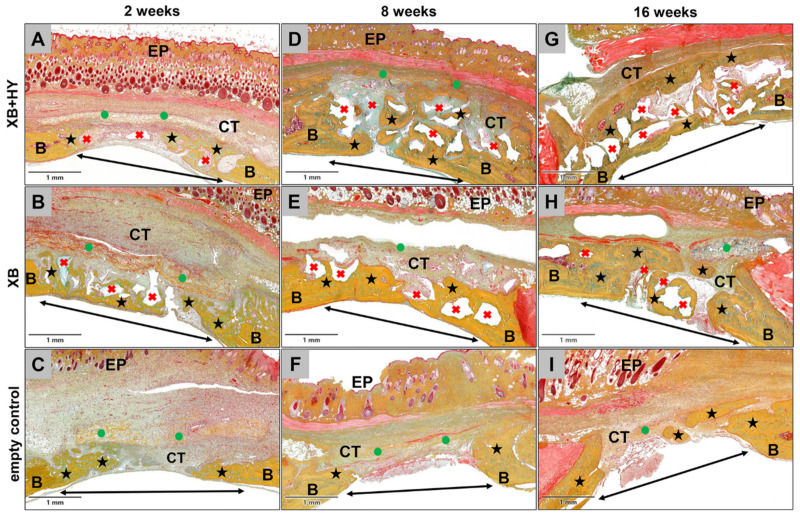
Representative overview of the bone healing process in the different study groups at 2 weeks (**A–C**), 8 weeks (**D–F**), and 16 weeks (**G–I**) *post implantationem.* Double arrow = bony implantation site, B = local bone, black asterisks = newly formed bone, red crosses = granules of the xenogeneic BSM, green circles = remnants of the collagen membrane, CT = connective tissue, EP = epidermis (Movat’s Pentachrome stainings, 100× magnifications, scalebars = 1 mm).

The histological analysis of the bone regeneration process revealed that only small amounts of newly formed bone were found in all study groups at 2 weeks post implantationem (Figure 1A–C). Thereby, it was observable that the bone formation was outgoing from the local bone tissue neighbored to the calvaria defects. In the grafted groups, the newly formed bone started to integrate the material granules neighbored to the local bone, indicating the osteoconductive properties of both materials (Figure 1A,B). However, most of the granules were integrated into a cell- and vessel-rich connective tissue at this early study time point (Figure 1A,B). In the empty control group, newly formed bone matrix seemed to be mainly associated with the regions of the dura mater (Figure 1C). Thus, new bone matrix was mainly located within the basal defect regions, while the remaining parts of the bone tissue defects were filled out by a cell- and vessel-rich connective tissue (Figure 1C).

At 8 weeks *post implantationem*, most parts of the former defect sites were filled by newly formed bone matrix that integrated most of the BSM granules in both material study groups (Figure 1D,E). Thereby, most of the granule surfaces were completely covered by bone matrix (Figure 1D,E). Only the central defect areas still contained cell- and vessel-rich connective tissue (Figure 1D,E). At this time point, most of the defect areas were still filled out by connective tissue in the empty control group, while the growth of new bone was slightly increased outgoing from the defect borders towards the central defect regions (Figure 1F).

At 16 weeks *post implantationem*, most of the former bone defects in both material groups were completely healed, integrating the granules of the xenogeneic BSM (Figure 1G,H). Thereby, nearly all surfaces of the material granules were covered by bone matrix at this study time point (Figure 1G,H). Only in a few cases were small spots observable, in which the bone regeneration process was not completed but was still in progress. In the control group, only approximately half of the defect areas regenerated, while in the other halves a cell- and vessel-rich connective tissue was still observed (Figure 1I). 

**Figure 2:**
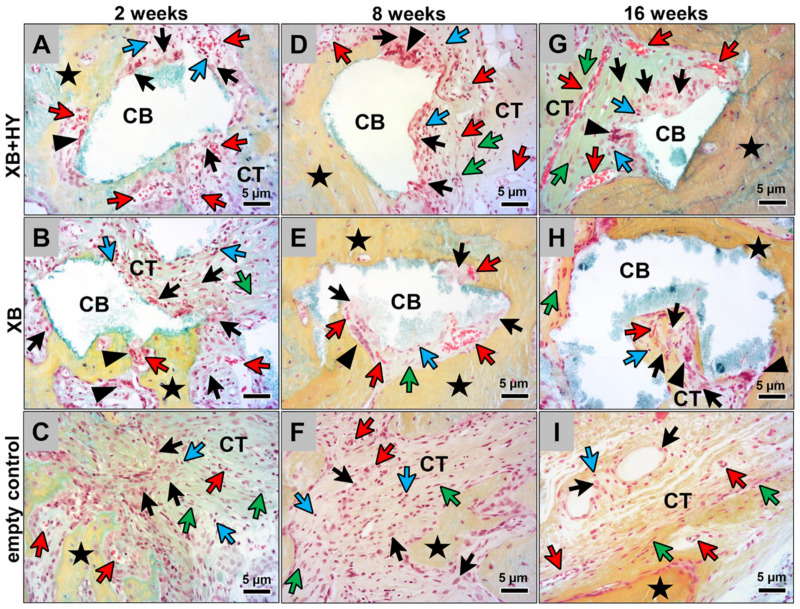
Exemplary histological images of the tissue responses to the BSM in the different study groups at 2 weeks (**A–C**), 8 weeks (**D–F**), and 16 weeks (**G–I**) *post implantationem.* At the material-tissue-interfaces, mainly macrophages in concert with single multinucleated giant cells were detectable. Within the surrounding connective tissue (CT) mainly macrophages, fibroblasts, and low numbers of lymphocytes were found in all study groups. Yellow arrows = macrophages, green arrowheads = multinucleated giant cells, violet arrows = lymphocytes, red arrows = blood vessels, blue arrows = fibroblasts, black stars = newly formed bone (Movat´s Pentachrome stainings, 400× magnifications, scalebars = 5 µm).

The histological analysis of tissue reactions at 2 weeks post implantationem showed that a moderate inflammatory reaction was found in all three study groups (Figure 2A–C). Thereby, intergranular connective tissue and also the connective tissue within the bone defects of the empty control group contained comparable numbers of different inflammatory cell types, i.e., mainly macrophages beside lower numbers of fibroblasts, lymphocytes, and a few granulocytes, as well as moderate numbers of small blood vessels (Figure 2A–C). In both material groups, comparable alignments of tissue reactions to the granules of the xenogeneic BSM were also found (Figure 2A,B). At the granule surfaces, mainly macrophages, as well as lower numbers of multinucleated giant cells (MNGCs), were detected (Figure 2A,B). Furthermore, no histological signs of the hyaluronate were found in the group treated with the hyaluronate containing BSM at this earl study time point (Figure 2A).

At 8 and 16 weeks *post implantationem*, the tissue reactions in all three study groups were also comparable (Figure 2D–I). The magnitude of the inflammatory tissue reactions had decreased in all groups, which was recognizable by lower numbers of inflammatory cells (Figure 2D–I). Within the intergranular tissue in the material groups and also within the connective tissue in the bone defects of the empty control group, mainly macrophages and fibroblasts, as well as low numbers of lymphocytes and only a few granulocytes, were still found (Figure 2D–I). Furthermore, the connective tissue in all groups showed a comparable vascularization, including mainly medium-sized vessels at both time points. In both material groups, mainly comparable numbers of macrophages and comparably lower numbers of MNGCs were detected at the material surfaces of the BSM granules that were covered by connective tissue (Figure 2D,E,G,H). Additionally, no signs of the hyaluronate were found at both time points in the group of the hyaluronate containing BSM (Figure 2D,G).

**Figure 3:**
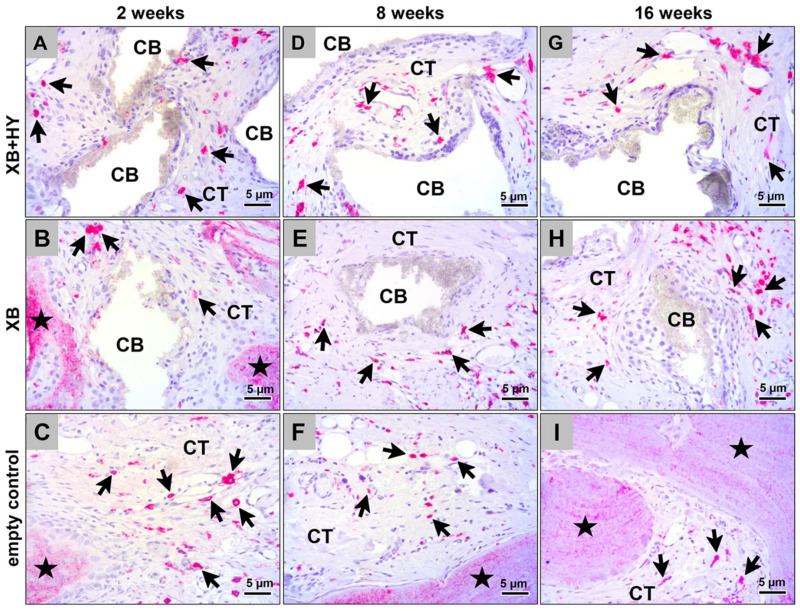
Representative histological images of the anti-inflammatory M2-macrophages within the subcutaneous connective tissue of the different study groups at 2 weeks (**A–C**), 8 weeks (**D–F**), and 16 weeks (**G–I**) *post implantationem.* Black arrows = M2-macrophages, black asterisk = newly formed bone, CB = granules of the xenogeneic BSM, CT = connective tissue, (CD163-immunostainings, 400× magnification, scalebars = 5 µm).

**Figure 4:**
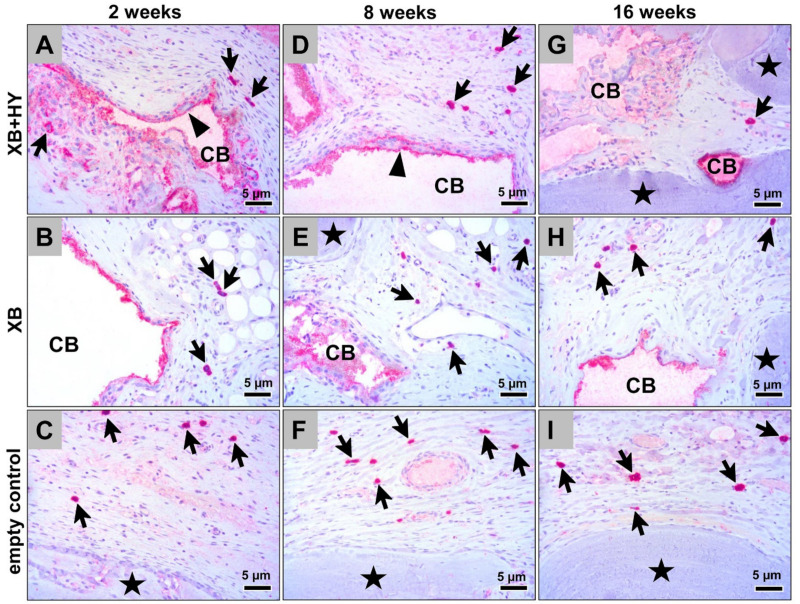
Representative histological images of the pro-inflammatory tissue response within the subcutaneous connective tissue of the different study groups based on immunohistochemical detection of the CD11c expression at 2 weeks (**A–C**), 8 weeks (**D–F**), and 16 weeks (**G–I**) post implantationem. Black arrows = M1-macrophages, black arrowheads = CD11c-positive multinucleated giant cells, black asterisk = newly formed bone, CB = granules of the xenogeneic BSM, CT = connective tissue, (CD11c-immunostainings, 400× magnification, scalebars = 5 µm).

The histological analysis of the occurrence of the pro- and anti-inflammatory macrophage subtypes showed that comparably high numbers of both cell types were detectable at all study time points and in all study groups (Figure 3 and Figure 4). Thereby, substantially higher numbers of anti-inflammatory macrophages were found in comparison to the numbers of pro-inflammatory subtypes (Figure 3 and Figure 4). Furthermore, it was observed that the material-adherent MNGCs only expressed the CD-11c molecule (Figure 4). 

**Figure 5:**
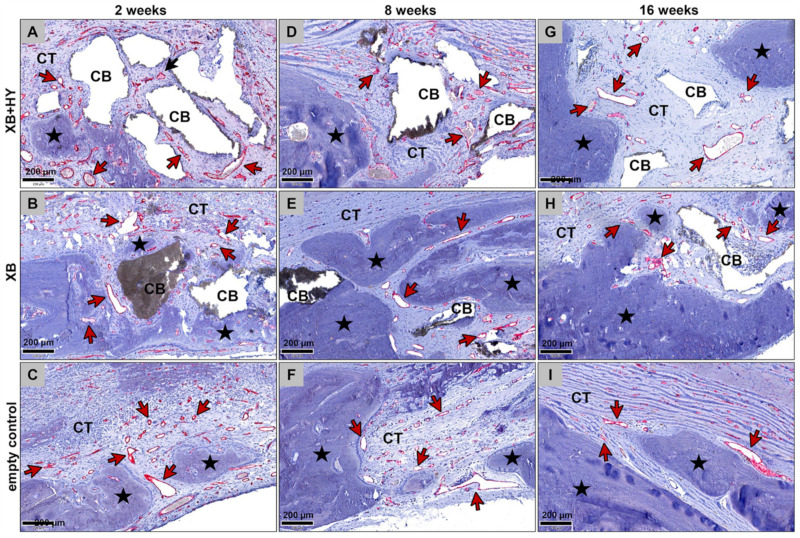
Representative overview of the vascularization pattern of the different study groups based on immunohistochemical detection of the CD31 expression at 2 weeks (**A–C**), 8 weeks (**D–F**), and 16 weeks (**G–I**) *post implantationem.* Red arrows = blood vessels, black asterisk = newly formed bone, CB = granules of the xenogeneic BSM, CT = connective tissue (CD31-immunostainings, 200× magnification, scalebars = 200 µm).

The histological analysis of the implant bed vascularization additionally showed that comparable numbers of vessels and vessel areas were detectable within the implantation beds in both BSM groups and the sham operation group at all study time points (Figure 5). The observations furthermore revealed that slightly decreasing vessel numbers were detectable over the study course in all study groups, while the strongest decrease was found in the sham operation group (Figure 5). Moreover, slight tendencies of decreasing vessel areas were observable in all study groups over the complete study period (Figure 5).

### 2.2. Results of the Histopathological Scoring

The histopathological scoring revealed at 2 weeks post implantationem that, within the bone defect areas including the implantation areas of both bone substitute materials and the sham operation group, the overall inflammation score was comparable (Table 1). At this early time point, the inflammatory tissue responses were mainly composed of polymorphonuclear cells, lymphocytes, and macrophages, which were found in comparable extents in all study groups. Only in the study groups of both bone substitute materials have comparably low numbers of multinucleated giant cells been found. Only in the sham operation group were very slight numbers of plasma cells detected. Furthermore, the neovascularization values were comparable in all groups (Table 1). In both material groups, comparable extents of very slight fibrosis were additionally found. Finally, no fatty infiltrate or necrosis was detectable in any of the study groups.

At 8 weeks post implantationem, the scoring showed that the overall inflammation within the defect areas and within the implantation areas were comparable in all study groups (Table 2). The inflammatory response was mainly composed of macrophages and lymphocytes at this time point without differences between the three study groups. Furthermore, comparable values of multinucleated giant cells were still found in both material groups, while significantly lower values were found in the sham operation group (Table 2). Comparably low values of polymorphonuclear cells and plasma cells were additionally detected in all study groups. The neovascularization was slightly higher in both material groups compared to the sham operation group, while fibrosis was slightly higher in this control group compared to the material groups (Table 2). Finally, very slight fatty infiltrate was found only in the sham operation group, while no necrosis was found in any of the study groups at this intermediate time point.

The scoring evaluation showed at 16 weeks post implantationem that comparable inflammation was found in all analyzed study groups (Table 3). Thereby, mainly macrophages and lymphocytes contributed to the inflammation in all study groups with similar values. Comparable low values of polymorphonuclear cells and plasma cells were found in all study groups. Furthermore, similar values of multinucleated giant cells were found in both material groups, while lower values were detected in the sham operation group. Additionally, similar values of neovascularization and fibrosis were detected in all study groups (Table 3). Finally, no or only slight values of fatty infiltration or necrosis were found in all study groups at this latest study time point. 

Based on the scoring values, the irritancy scores were calculated. This analysis showed that XB+HY had an average treatment irritancy score of 12.75, and the Control Articles (XB) had an average treatment irritancy score of 9.79 at 2 weeks post implantationem (Table 4). Thus, the overall irritancy score for XB+HY plus was 2.96, and this BSM was considered to be non-irritant at this early study time point. Additionally, a treatment irritancy score of 9.40 was calculated in the sham operation group.

This analysis furthermore showed that XB+HY had an average treatment irritancy score of 12.93, and the Control Article (XB) had an average treatment irritancy score of 9.79 at 8 weeks post implantationem (Table 4). Thus, the overall irritancy score for XB+HY was 2.18, and this BSM was considered to be a non-irritant at this study time point. At this time point, the treatment irritancy score within the sham operation group was 8.67 (Table 4).

The analysis revealed at 16 weeks post implantationem that XB+HY had an average treatment irritancy score of 11.50, and the Control Article (XB) had an average treatment irritancy score of 11.33 (Table 4). Thus, the overall irritancy score for XB+HY was 0.17, and this BSM was considered to be a non-irritant at this study time point. At this time point, the treatment irritancy score within the sham operation group was 9.00 (Table 4).

### 2.3. Histomorphometrical Analysis

#### 2.3.1. Analysis of Bone Regeneration

**Figure 6:**
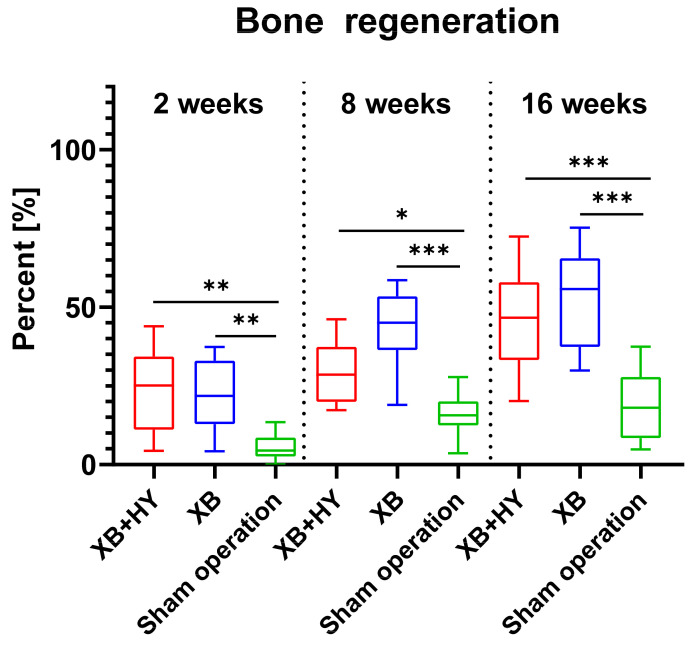
Results of the histomorphometrical analysis of bone regeneration presented as mean and standard deviation (* *p* < 0.05, ** *p* < 0.001, *** *p* < 0.0001, * = inter-individual significances).

The histomorphometrical analysis of bone regeneration showed that comparable amounts of newly formed bone were detected in the study groups of the hyaluronate containing BSM and the control xenogeneic BSM at 2 weeks post implantationem (Table 5 and Figure 6). The values in both groups were significantly higher compared to the values in the empty control group (** *p* < 0.001) (Table 5 and Figure 6).

At 8 weeks post implantationem, the histomorphometrical analysis of bone regeneration showed that still comparable amounts of newly formed bone were found in the study groups of the hyaluronate containing BSM and the control xenogeneic BSM (Table 5 and Figure 6). The amounts in both groups were found to be significantly higher compared to the values in the sham operation group (* *p* < 0.001 and *** *p* < 0.0001) (Table 5 and Figure 6). 

At 16 weeks post implantationem, the histomorphometrical analysis of bone regeneration revealed still comparable amounts of newly formed bone tissue in the study groups of the hyaluronate containing BSM and the control xenogeneic BSM that both differed significantly from the lower values in the empty control group (*** *p* < 0.0001) (Table 5 and Figure 6). 

The analysis of the intraindividual significances showed that the values at 16 weeks differed highly significantly from that found at 2 weeks post implantationem in the group of the hyaluronate containing BSM (●● *p* < 0.001) (not graphed). In the group of the control BSM, the values at 8 and 16 weeks post implantationem differed highly significantly from the amounts of newly formed bone detected at 2 weeks (●● *p* < 0.001 and ●●● *p* < 0.0001). Additionally, the values at 8 and 16 weeks post implantationem differed highly significantly from those measured at 2 weeks in the sham operation group (●● *p* < 0.001 and ●●● *p* < 0.0001).

#### 2.3.2. Analysis of the Immune Response

**Figure 7:**
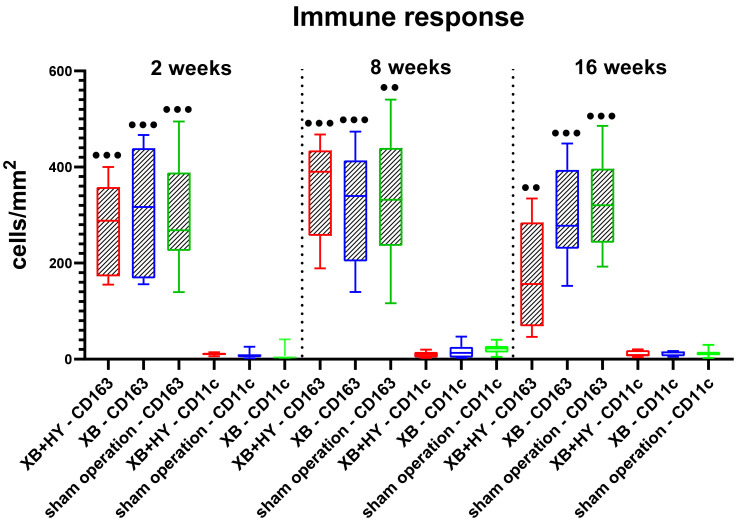
Results of the histomorphometrical analysis of the immune response represented by the numbers of CD163-positive anti- and CD11c-proinflammatory macrophages within the implantation beds, presented as mean and standard deviation (●● *p* < 0.01 and ●●● *p* < 0.001, ● = intra-individual significances).

The histomorphometrical analysis of the occurrence of M1- and M2-macrophages showed that comparable numbers of both subtypes were found at every study time point within the implantation beds of the hyaluronate containing BSM, the control BSM, and the sham operation group (Table 6 and Figure 7). Moreover, the numbers of anti-inflammatory cells were highly significantly higher in all groups and at all study time points compared to the numbers of pro-inflammatory macrophages (●● *p* < 0.001 and ●●● *p* < 0.0001) (Figure 7).

#### 2.3.3. Analysis of the Implant Bed Vascularization 

**Figure 8:**
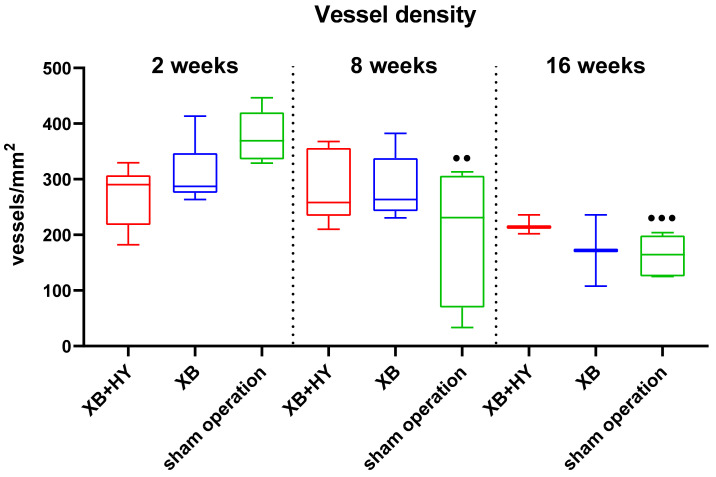
Results of the histomorphometrical analysis of the vessel densities within the implantation beds presented as mean and standard deviation (●● *p* < 0.01 and ●●● *p* < 0.001, ● = intra-individual significances).

The histomorphometrical analysis of the implantation bed vascularization showed that comparable vessel numbers have been found at every study time point within the implantation beds of the hyaluronate containing BSM, the control BSM, and the sham operation group (Table 7 and Figure 8). Moreover, the vessel numbers decreased significantly compared to the numbers at 2 weeks post implantationem in the sham operation group (●● *p* < 0.01 and ●●● *p* < 0.001) (Figure 8).

**Figure 9:**
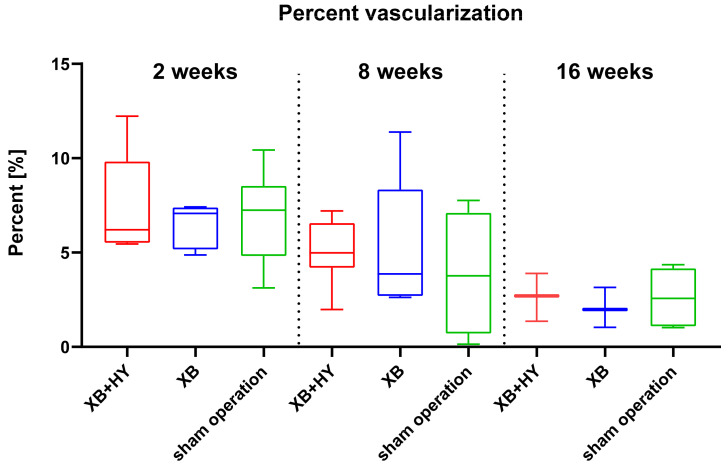
Results of the histomorphometrical analysis of the vascularization within the implantation beds presented as mean and standard deviation (in percent (%)).

The histomorphometrical analysis of the percent vascularization showed that no differences between the study groups were detected at any of the study time points (Table 8 and Figure 9). Furthermore, no intraindividual differences were found between the different study time points in the three study groups. However, similar tendencies towards a decrease of the percentage of vascularization were observed in all study groups up to 16 weeks post implantationem (Table 8 and Figure 9).

## 3. Discussion

Bone grafts of paste-like consistency are more and more used in hard tissue regeneration, not only in the dental field but also in orthopedics or traumatology. It has been shown that their applicability ranges from sinus augmentation procedures over bone cyst fillings to jawbone regeneration procedure [38]. Thereby, they allow for more precise application, even into deeply enclosed defects and for the repair of irregularly shaped lesions [39]. 

Mainly, bone graft pastes based on calcium phosphate (CaP) granules combined with water-binding molecules such as collagen, cellulose, or hyaluronate (HY) are used in dentistry [39]. All of them are based on synthetic CaP granules composed of pure CaP phases such as hydroxyapatite (HA) or β-tricalcium phosphate (β-TCP) or mixtures of both compounds called biphasic BSM [38]. In this context, it has been shown that these BSM have their own indication area in which their application leads to the desired regenerative outcome [18,40]. Thereby, its regenerative properties are mainly depending on the integrated CaP granules. Especially in the case of synthetic BSM, it has been shown that they undergo degradation via (a) dissolution to calcium and phosphate ions in the organism and (b) cellular degradation or phagocytosis mediated by macrophages and multinucleated giant cells (MNGCs) [41]. Their degradation pattern depends on the phase composition, and a degradation pattern that is correlating with simultaneous bone ingrowth has mainly appeared in the case of biphasic BSM [42]. Thus, it can be concluded that the application of such BSM are mainly indicated in regenerative procedures that do not need a permanent maintenance of an osteoconductive basis such as jawbone reconstructions in the case of extraction sockets, after bone cysts, furcation defects, or other (intra-) osseus defects [43]. However, indications that require permanent bone maintenance or volume stability, such as sinus augmentation procedures or jawbone regeneration even in the case of larger defects or bone resorption locations in the case of (long-term) edentulous patients—particularly if this condition might be strengthened by comorbidities such as osteoporosis or bone cancer —need the application of BSM with a prolonged degradation behavior. Xenogeneic BSMs such as Bio-Oss^®^ or cerabone^®^ have been shown to provide this resorption profile as they have been found within their implantation beds several years after their implantation. Interestingly, the molecular basis of their biodegradation pattern has already been revealed in different studies, showing that this material type does induce a low grade of material-associated inflammation and especially of low numbers of multinucleated giant cells. Thereby, a broad variety of clinical studies revealed their good bone regeneration capacities [14,44].

Xenogeneic BSMs are mainly applied in the form of granules, and also in the form of blocks [45]. However, there is not yet a BSM available combining xenogeneic bone substitute granules with hyaluronic acid. The base principle of this two-component material class is to mix BSM granules with a hydrogel composed of minimally one water-binding polymer to increase the clinical handling [39]. In particular, the extracellular matrix protein hyaluronic acid (HY)—and even high molecular weight HY (HMWHY)—has gained importance as a component of actual BSM [46]. Besides its water-binding properties, it is believed to trigger the bone regeneration process by influencing underlying molecular processes such as osteoblast differentiation, proliferation, and migration [47]. Additionally, it has been revealed that HY can increase implant bed vascularization based on its influence on endothelial cells and angiogenesis [48]. Not least, the HY addition is believed to have an influence on macrophage polarity towards an M2-phenotype, contributing to an overall anti-inflammatory alignment of the material-related foreign body reaction and an improved bone tissue regeneration [31]. Finally, it has been reported that its addition might allow for a special integration pattern in accordance with the process of Guided Bone Regeneration (GBR) [28,49]. 

The present in vivo study was conducted by analyzing the (inflammatory) tissue reaction in combination with the bone healing capacities of a newly developed BSM based on an xenogeneic BSM combined with HMWHY. Established in vivo methods including specialized histological workup processes, (immuno-) histological staining procedures, and both histopathological and histomorphometrical analysis protocols according to the DIN EN ISO 10993-6 were applied after implantation of the hyaluronate containing BSM using the calvaria model in Wistar rats. Implantations of the xenogeneic BSM not containing HMWHY and sham operations were used as control groups.

Altogether, the results of the present study show that the HY allows for an optimal material-associated bone tissue healing comparable to the control xenogeneic BSM. The added HY seems to be degraded within a very short time period of less than 2 weeks so that the remaining BSM granules allow for a gradual osteoconductive bone regeneration. Additionally, no differences between the inflammatory tissue reactions in both material groups and the sham operation group were found.

The results of the study, including not only the histological observations but also the histomorphometrical measurements, initially showed that comparable bone regeneration values were found in the analyzed xenogeneic BSM group and the group of the control xenogeneic BSM. This result shows that the initially implanted HMWHY seemed not to influence the bone growth process. Thus, it is conceivable that the HMWHY served as a hydrogel component during the implantation but was degraded prior to having a molecular influence on the bone regeneration or associated processes. In this context, Snetkov et al. described the degradation pattern of HY—although only poor knowledge is available about the degradation times [50]. Within tissues, HY is generally degraded via enzymatic hydrolysis of hyaluronidases (HYALs), and even HYAL1 and HYAL2 are considered major HA-degrading enzymes [51]. Moreover, it was reported in different studies that HMWHY was finally degraded within 24–48 h [50]. Thus, it is conceivable that the HMWHY present in the analyzed BSM did not have any effect on the material-associated tissue reactions. Furthermore, it was shown in another study series, including in vitro, in vivo, and clinical study parts that combinatorically analyzed the tissue reactions and the (bony) integration behavior of a bone paste based on β-TCP granules with a hydrogel combining HY and methylcellulose, showed that this material underwent continuous degradation from the periphery towards the core [24]. Additionally, the combination of the three biocompatible materials into one material enabled modification of the tissue reaction to the implant and resulted in a longer in vivo lifetime than that of β-TCP granules alone. In addition, this combination increased the vascularization of the implantation bed. While these study results attributed the observed reaction pattern to the added HY, the results of the present study led to the conclusion that it might rather be traced to the methylcellulose. This assumption cannot completely be confirmed as the aforementioned study series did not include a respective control group without methylcellulose. Another in vivo study by Sieger et al. analyzed the tissue reactions and the bone regeneration to a synthetic biphasic BSM combined with two different amounts of HMWHY [32]. Again, no differences in the bone healing, even compared to the control group (without HY), were detected, which underlines the present results.

The results of the present study showed that, in all study groups, an increasing bone volume was detected over the study period, which was expectable. Moreover, significantly higher bone regeneration was found in both material groups, substantiating the good osteoconductive properties and the good biocompatibility of the xenogeneic BSM, which has already been examined and confirmed in different other studies [24,32,52,53]. Additionally, the analysis showed that the values of newly formed bone tissue in the sham operation group were significantly lower compared to both material groups, which underlines the idea that the calvaria implantation site reached a critical size. Thus, bone regeneration cannot take place to the same extent as in the groups containing the osteoconductive BSM. This result substantiates the regenerative capacity of the xenogeneic bone substitute.

The results of (a) the histopathological analysis, (b) the scoring, and (c) the histomorphometrical analysis of the immune response via detection of M1- and M2-macrophages combinatorically showed that the added HMWHY did not have an effect on the cellular or inflammatory reactions to the xenogeneic BSM. This result also supports the aforementioned assumption of the fast degradation of the added HMWHY. Interestingly, the analysis of the M1- and M2-macrophage induction additionally showed that none of the materials, i.e., the hyaluronate containing BSM and the control BSM, induced significant differences in view of the macrophage subtypes found in the sham operation group. Moreover, no differences in cell numbers were found while conducting the histopathological scoring. Even in view of the heavily discussed topic of potential immune responses to natural biomaterial, such as the analyzed xenogeneic BSM, the presented results show that the xenogeneic BSM did not seem to evoke an immune response contrary to the sham operation group [54,55,56]. In this context, Kačarević et al. stated in a review article that the xenogeneic BSM analyzed in the present study is treated with temperatures above 1200 °C, which has been reported to safely reduce the risk of pathogen transmission and eliminates cell or protein content. Thus, the present data substantiate the previous results that are also proved in a new study by Barbeck et al. analyzing the systemic and local immune response to 15 commercially available BSMs and allogeneic bone grafts after subcutaneous implantation for up to 90 days [57] (manuscript submitted). These data reveal that none of the materials induced increased levels of 14 measured cytokines from the peripheral blood, while only slight local effects onto macrophage responses were found. 

Interestingly, the results of these study parts clearly showed that, in all study groups, more M2-macrophages compared to their M1-subtype were found. In combination with data from bone growth measurements, it can be concluded that both analyzed biomaterials provide an excellent biocompatibility, creating a microenvironment optimally suitable for bone tissue healing [58]. However, it must be mentioned that the analysis of the local effects of biomaterials, such as the analyzed BSM, which are only based on the macrophage counting, may only provide limited data. In combination with the histopathological scoring data, more information is provided, but it still shows some limitations. This is because the scoring that is based on the ISO-norm 10993 does not include relevant cell types such as T- or B-lymphocytes or even mast cells. Thus, a revision of the ISO-norm including more cell types and also a differentiation of different cell subtypes may be necessary. This approach may not only help to introduce safer materials into the market but also help to develop a next generation of BSM that modulate the immune response to improve (bone) tissue regeneration. However, it must be mentioned that most of the studies analyzing tissue reactions to biomaterials such as the analyzed BSM include less information than presented in the actual study.

Finally, the measurements of the implant bed vascularization showed a consistently comparable vessel density in all study groups, while only a significant decrease up to 16 weeks post implantationem was found in the sham operation group. The analysis of the percent vascularization did not show any significant differences between or within the different groups over the study period, while in all groups a decreasing tendency was detectable up to the end of the study period. These data additionally lead to the conclusion that neither the analyzed hyaluronate containing BSM nor the control xenogeneic BSM evoked pronounced inflammatory tissue reactions, as already shown by the other study parts. This assumption is based on the fact that it has been shown that both macrophages and multinucleated giant cells (MNGCs) are expressing pro-angiogenic molecules such as the vascular endothelial growth factor (VEGF) that has a major influence on the implantation bed vascularization, even in the case of biomaterials inducing strong tissue reactions [59]. Different studies have revealed that both vascularization parameters are especially dependent on the numbers of MNGCs induced by a biomaterial such as synthetic BSM [24,37,47,59,60,61]. The results of the present study showed in conformity with other preclinical studies analyzing the tissue reactions to the same xenogeneic material implanted within the subcutaneous tissue and also after its clinical application for sinus augmentation that both BSMs induced a low tissue reaction pattern, including low numbers of MNGCs [12,36]. Thus, a correlating low vascularization pattern was expectable and has been shown in the present study. Interestingly, both the vessel density and the percentage of vascularization differed from that detected in the previous study using the subcutaneous implantation model that measured a significant implant bed vascularization [12]. This data reflects the reported differences of the tissue reactions using different implantation models [62]. In this context, it has already been assumed that the different micromilieus lead to differences in measurement data such as the observed vascularization pattern. Thus, data gained via the subcutaneous implantation model—especially in the case of BSM—may not represent the physiological conditions existing within the “bone tissue compartment”. However, data gained by this preclinical implantation model can initially give basic insights into the tissue reaction pattern to biomaterials such in vitro cytocompatibility analyses that are also proposed by the ISO guidelines. Thus, further studies have to prove the comparability of data won by different implantation models—even with regard to different biomaterials for different clinical applications.

In summary, the results of the present study show that the newly developed bone grafting material composed of xenogeneic BSM granules combined with HMWHY provides an excellent biocompatibility and good osteoconductive properties that are fully comparable to the pure BSM granules whose performance has already been tested in a broad variety of preclinical and clinical studies [14,44,63]. Moreover, the scoring, in combination with the molecular biological analyses, showed that this hyaluronate containing BSM induced a similar tissue reaction pattern comparable to the control BSM. Moreover, similar M1- and M2-macrophage numbers were also found in the sham operation group. These data show that the added HMWHY did not induce effects on the bone healing process or on the inflammatory tissue reaction or on the implantation bed vascularization. It also shows that both BSM did not evoke pronounced inflammatory tissue reactions substantiating their purity and tissue compatibility.

## 4. Material and Methods

### 4.1. Biomaterials

All biomaterials analyzed in the present study were kindly provided by botiss biomaterials GmbH (Zossen, Germany).

#### 4.1.1. Cerabone^®^

The xenogeneic BSM cerabone^®^ (XB, botiss biomaterials GmbH, Zossen, Germany) is obtained from the femoral heads of cattle from registered slaughterhouses in New Zealand and Germany. The potentially immunogenic components are removed in a multi-step-process to ensure its safe application [11]. Thereby, the bovine bone raw material undergoes a sophisticated three-step heating, which is free of chemical additives and includes a final high temperature treatment at more than 1200 °C. After the purification processes the bone substitute material is packed and sterilized. For the present in vivo study cerabone^®^ granules with a particle size of 0.5–1 mm were used.

#### 4.1.2. Cerabone^®^ plus

Cerabone^®^ plus (XB+HY, botiss biomaterials GmbH, Zossen, Germany) is a combination of cerabone^®^ granules and high molecular weight sodium hyaluronate. The bone substitute material is provided dry and has to be hydrated before use. Upon hydration with saline or blood, it forms a malleable bone grafting material, which facilitates the application and reduces particle distribution in the augmentation area. For the present study, cerabone^®^ plus with cerabone^®^ granules in the size of 0.5–1 mm were used. 

### 4.2. In Vivo Study Design, Implantation and Explantation Procedure

The study included 42 male 10–12-week-old Wistar rats with an approximate weight of 220–240 g, which were randomly allocated in three different study groups. Thus, each group consisted of 21 animals with 7 animals per study time point (*n* = 7) (Table 9). Explantation was timed at 2, 8, and 16 weeks (Table 9). Prior to the study, the in vivo experiments were authorized by the local Ethical Committee of the Faculty of Medicine (University of Niš, Serbia). Approval of the Local Ethical Committee (Faculty of Medicine, University of Niš, Niš, Serbia) was based on decision number 323-07-00073/2017-05/7 of the Veterinary Directorate of the Ministry of Agriculture, Forestry and Water Management of the Republic of Serbia (date of approval: 22/02/2017). Animal welfare included animal housing and standard operative care in compliance with standardized guideline for animal experiments (e.g., water ad libitum, artificial light-dark cycle of 12 h each, controlled temperature and humidity, regular rat pellets). The animal welfare was conducted and ensured by appropriately qualified and trained staff at the Faculty of Medicine (University of Niš, Serbia). Standard veterinary medical care was provided in this study, and only healthy animals were selected for implantation.

To evaluate the tissue response to the newly developed BSM after implantation, three groups were set (Table 9).

#### Calvaria Implantation

After an acclimation period of 1 week, the surgical interventions for implantation of the biomaterials according to the study plan were conducted as previously described. Prior to the implantation, intraperitoneal anesthesia with 10 mL ketamine (50 mg/mL) with 1.6 mL Xylazine (2%), was performed, and animals were shaved and disinfected. Bilateral cranial defects (8 mm diameter) were created using a trephine bur (GC, Tokyo, Japan). Left-sided defects were filled with equal amounts of the BSM, while the right-sided defects served as controls without biomaterial insertion (control group). Both defects were covered via a collagen membrane (Jason^®^ membrane, botiss biomaterials GmbH, Zossen, Germany) and sutured. After the predefined healing periods (2, 8, and 16 weeks), the animals were euthanized by means of euthasol (400 mg/mL), followed by the extraction of the implantation area and subsequent histological workup. The explanted tissues were initially fixed using 4% formalin solution for 48 h and were then stored in phosphate-buffered saline (PBS) at 4 °C until further histological preparation.

### 4.3. Sample Preparation and Staining Procedures

By means of a diamond-bandsaw (Diamond-bandsaw Makro, EXAKT Advanced Technologies GmbH, Norderstedt, Germany) the calvarial explants were cut into segments that contained both bone defects, i.e., the left and the right defect. For further histological processing, the explants were placed in embedding cassettes (Histosette^®^, VWR, Darmstadt, Deutschland). Automatic dehydration was performed in a series of increasing alcohol concentrations (80%, 96%, 100%) to prepare the tissue for following plastic embedding in Technovit 9100 (Technovit 9100, Kulzer GmbH, Hanau, Germany) After dehydration, stepwise immersion at 4 °C with Technovit 9100 medium using different infiltration solutions (pre-infiltration, infiltration I + II with same composition) was conducted. Afterwards, the polymerization solution was prepared according to the operation instructions. The explants were orientated on the saw-edges and placed on the bottom of rolled rim bottles (rolled rim bottles with snap-on lid (VWR, Darmstadt, Germany), subsequently followed by pouring with the polymerization mixture. To avoid exposition of oxygen and therefore occurrence of irregular polymerization, the bottles were sealed hermetically and immediately stored at −20 °C until the liquid Technovit 9100 was completely polymerized and hardened. Subsequently, the tissue blocks were trimmed into shape by means of a grinding machine (EcoMet 30, Buehler, Esslingen, Germany). Sections with a thickness of 4–6 µm were prepared using a rotation microtome (CUT4060E, microTec GmbH, Walldorf, Germany). Histochemical and immunohistochemical staining was performed using specialized methods, as previously published. Four sections of every tissue explant were used for hematoxylin and eosin (H&E), Masson–Goldner, Movat’s Penatchrome, and Heidenhain’s Azan trichrome stainings. Two additional sections were used for immunohistochemical staining. Briefly, antibodies for detection of pro- and anti-inflammatory macrophage subtypes, i.e., integrin alpha x (CD11c) (abx231412, Abbexa Ltd, Milton, United Kingdom) and hemoglobin scavenger receptor (CD163) (ab182422, abcam, Cambridge, UK), were used to assess the immunological tissue response. Initially, the slides were treated with TRIS-EDTA pH 9 for 20 min in a steamer at 96 °C, followed by equilibration using cold wash buffer. Before the incubation with the respective first antibody for 60 min at room temperature, a blocking step with protein blocking solution for 10 min was conducted. Final detection of the antigen was caused by incubation with the biotinylated secondary antibody for 15 min, subsequently followed by application of the streptavidin–alkaline–phosphatase conjugate and the permanent alkaline phosphatase (AP)-red chromogen. Finally, counterstaining was performed using Mayer’s hemalum solution (Merck KGaA, Darmstadt, Germany). Unless otherwise stated, all solutions and reagents were purchased from Zytomed Systems (Berlin, Germany).

### 4.4. Histopathological & Histomorphometrical Analysis

To evaluate the inflammatory tissue response and osseointegration, the groups were compared histologically and based on histopathological scoring system according to the respective DIN ISO norm 10993-6 [64]. Thereby, XB+HY (group 1) was compared with XB (control group) to evaluate the effect of the addition of sodium hyaluronate.

The histology sections were evaluated for a number of parameters, evaluating safety and efficacy. The sections were analyzed and graded according to cell type and responses. Safety was evaluated following the irritancy/reactivity grading scheme adapted from the ISO 10993-6 Annex E (Table 10).

Afterwards, irritancy/reactivity scores based on ISO 10993-6 derived from the parameters listed in Table 10 were calculated as follows for each defect (Table 11):

The irritancy score for each test or control treatment was then calculated by averaging the irritancy scores of all test or control defect sites for each treatment, respectively. Each irritancy/reactivity score was calculated as follows:

- Test Article Irritancy Score—Control Article Irritancy Score = Irritancy/Reactivity score;
- If the result was a negative number, the Irritancy/Reactivity Score was considered to be 0.0.

Additionally, the macrophage numbers, the bone growth, and the implantation bed vascularization were examined histomorphometrically using specialized digital methods, as previously described [64]. For the histomorphometrical analysis, the regions of interest, including newly formed bone, remaining BSM, and soft-tissue cavities, were digitized with a specialized scanning microscope (M8, precipoint, Munich, Germany). For comparative measurements of the tissue distribution, the respective areas of newly built bone, remaining BSM, and connective tissue within the implantation area were measured by manually marking the different tissue parts using the open-source software ImageJ. The tissue fractions were calculated by calculating the respective percentage of the fraction area in relation to the total implant area. The occurrence of pro- and anti-inflammatory macrophages within the implant beds was also determined with the ImageJ software using a specially developed plugin, as already described by Lindner et al. [64]. For this measurement step, the workflow-plugin allowed us to calculate the respective cell densities via relation to the total implant area (cells/mm^2^). For analysis of the implantation bed vascularization, the vessels were marked based on a newly developed plugin for ImageJ [Linder et al., manuscript in preparation] and related to the total implant area as vessel density (vessels/mm^2^) and as percent vascularization (total vessel area in relation to the total implant area, in %). 

### 4.5. Statistical Analysis

An analysis of variance (ANOVA) was used via the GraphPad Prism 8.0 software (GraphPad Software Inc., La Jolla, CA, USA), followed by an LSD post-hoc test for statistical analysis of the qualitative data won via histomorphometry. Both inter- (*) and intra-individual (●) significances were calculated and designated as significant if the *p*-values were less than 0.05 (*/● *p* ≤ 0.05), and highly significant if the *p*-values were less than 0.01 (**/●● *p* ≤ 0.01) or less than 0.001 (***/●●● *p* ≤ 0.001). Finally, the data were shown as mean and standard deviations. 

## Figures and Tables

**Table 1:** Results of the scoring evaluation at 2 weeks post implantationem.

Parameter	Mean ± SD—Inflammation and Inflammatory Cell Types at 2 Weeks								
	XB+HY			XB			Sham Operation		
Inflammation Associated with Biomaterial /Region of Biomaterial	1.75	±	0.42	1.64	±	0.24	1.3	±	0.45
Polymorphonuclear Cells	1.08	±	0.49	0.57	±	0.19	1	±	0.61
Lymphocytes	1.42	±	0.49	1.07	±	0.45	1	±	0.35
Plasma Cells	0	±	0	0	±	0	0.5	±	0.5
Macrophages	2	±	0.32	1.64	±	0.38	1.4	±	0.42
Giant Cells	1.25	±	0.42	1	±	0.71	0	±	0
Neovascularization	1.17	±	0.41	1.14	±	0.24	1.6	±	0.22
Fibrosis	0.08	±	0.2	0.07	±	0.19	0	±	0
Fatty infiltrate	0	±	0	0	±	0	0	±	0
Necrosis	0	±	0	0	±	0	0	±	0

**Table 2:** Results of the scoring evaluation at 8 weeks post implantationem.

Parameter	Mean ± SD—Inflammation and Inflammatory Cell Types at 8 Weeks								
	XB+HY			XB			Sham Operation		
Inflammation Associated with Biomaterial /Region of Biomaterial	1.86	±	0.48	1.67	±	0.26	1.4	±	0.22
Polymorphonuclear Cells	0.71	±	0.39	0.5	±	0	0.5	±	0
Lymphocytes	1.21	±	0.27	1.17	±	0.26	1.25	±	0.42
Plasma Cells	0.07	±	0.19	0.25	±	0.27	0.33	±	0.26
Macrophages	2.14	±	0.24	1.75	±	0.27	1.5	±	0
Giant Cells	1.64	±	0.24	1	±	0.45	0.17	±	0.26
Neovascularization	1.29	±	0.39	1.33	±	0.41	0.75	±	0.42
Fibrosis	0.07	±	0.19	0.08	±	0.2	0.33	±	0.41
Fatty infiltrate	0	±	0	0	±	0	0.08	±	0.2
Necrosis	0	±	0	0	±	0	0	±	0

**Table 3:** Results of the scoring evaluation at 16 weeks post implantationem.

Parameter	Mean ± SD—Inflammation and Inflammatory Cell Types at 16 Weeks								
	XB+HY			XB			Sham Operation		
Inflammation Associated with Biomaterial / Region of Biomaterial	1.3	±	0.41	1.83	±	0.26	1.17	±	0.26
Polymorphonuclear Cells	0.75	±	0.27	0.5	±	0	0.67	±	0.26
Lymphocytes	1.17	±	0.26	1.5	±	0.45	1	±	0.32
Plasma Cells	0	±	0	0.25	±	0.42	0.5	±	0.32
Macrophages	1.75	±	0.27	1.92	±	0.2	1.17	±	0.26
Giant Cells	1.25	±	0.42	0.83	±	0.41	0.17	±	0.26
Neovascularization	1.25	±	0.42	1.17	±	0.26	1.33	±	0.52
Fibrosis	0.42	±	0.58	0.17	±	0.26	0.5	±	0
Fatty infiltrate	0	±	0	0	±	0	0.17	±	0.41
Necrosis	0	±	0	0	±	0	0	±	0

**Table 4:** Irritancy scores and irritancy status at 2, 8, and 16 weeks post implantationem.

	Study Group	Treatment Irritancy Score	Overall Irritancy Score	Irritant Status
**2 Weeks**	XB+HY	12.75	2.96	non-irritant
	XB	9.79	9.79	-
	sham operation	9.40	9.40	-
**8 Weeks**	XB+HY	12.93	2.18	non-irritant
	XB	10.75	10.75	-
	sham operation	8.67	8.67	-
**16 Weeks**	XB+HY	11.50	0.17	non-irritant
	XB	11.33	11.33	-
	sham operation	9.00	9.00	-

**Table 5:** Amounts of newly formed bone tissue in the different study groups and at the three study time points (in %).

Study Group	2 Weeks	8 Weeks	16 Weeks
XB+HY	23.4 ± 13.9	29.3 ± 10.3	46.1 ± 22.2
XB	22.1 ± 12.1	43.6 ± 13.5	53.6 ± 15.8
Sham Operation	5.7 ± 3.6	16.2 ± 6.2	19.2 ± 10.3

**Table 6:** Numbers of anti- and pro-inflammatory macrophages within the calvarial defect sites of the different study groups (cells/mm^2^).

Study Group	2 Weeks		8 Weeks		16 Weeks	
	CD163+	CD11c+	CD163+	CD11c+	CD163+	CD11c+
XB+HY	276.9 ± 96.5	10.7 ± 2.8	359.5 ± 99.9	9.7 ± 6.4	172.7 ± 115.0	11.6 ± 6.8
XB	326.2 ± 128.1	9.5 ± 14.1	318.7 ± 122.4	16.5 ± 14.9	298.2 ± 103.0	11.8 ± 5.2
sham operation	300.7 ± 108.2	8.2 ± 5.1	331.9 ± 117.8	21.2 ± 9.9	323.7 ± 86.9	12.5 ± 6.4

**Table 7:** Vessel densities within the calvarial defect sites of the different study groups (vessels/mm^2^).

Study Group	2 Weeks	8 Weeks	16 Weeks
XB+HY	270.3 ± 54.22	281.4 ± 63.72	217.3 ± 17.07
XB	309.2 ± 54.87	285.0 ± 58.97	171.9 ± 64.16
sham operation	377.2 ± 45.61	202.2 ± 127.0	162.5 ± 36.93

**Table 8:** Vascularization within the calvaria defect sites of the different study groups (in percent (%)).

Study Group	2 Weeks	8 Weeks	16 Weeks
XB+HY	7.44 ± 2.69	5.07 ± 1.77	2.65 ± 1.27
XB	6.51 ± 1.15	5.19 ± 3.62	2.06 ± 1.06
sham operation	6.88 ± 2.47	3.86 ± 3.30	2.61 ± 1.54

**Table 9:** Overview of the study groups displaying the experimental animals per group and time point.

	Group 1 XB+HY	Group 2 XB	Group 3 Empty Defect
**2 weeks**	7	7	14
**8 weeks**	7	7	14
**16 weeks**	7	7	14
**Number per study group**	21	21	--
**Total number**	42 experimental animals		

**Table 10:** Histologic Evaluation System for Irritancy/Reactivity—Cell Type/Response.

Response	Score (phf = per high powered (×400) Field)				
	0	1	2	3	4
Polymorphonuclear cells	0	Rare, 1–5/phf *	6–10/phf	Heavy infiltrate	Packed
Lymphocytes	0	Rare, 1–5/phf	6–10/phf	Heavy infiltrate	Packed
Plasma cells	0	Rare, 1–5/phf	6–10/phf	Heavy infiltrate	Packed
Macrophages	0	Rare, 1–5/phf	6–10/phf	Heavy infiltrate	Packed
Giant cells	0	Rare, 1–2/phf	3–5/phf	Heavy infiltrate	Packed
Necrosis/osteolysis	0	Minimal	Mild	Moderate	Marked
Neovascularization	0	Minimal capillary proliferation focal, 1–3 buds	Groups of 4–7 capillaries with supporting fibroblastic structures	Broad band of capillaries with supporting structures	Extensive band of capillaries with supporting fibroblastic structures
Fibrocytes/fibroconnective tissue, fibrosis	0	Narrow band	Moderately thick band	Thick band	Extensive band
Fatty infiltrate	0	Minimal amount of fat associated with fibrosis	Several layers of fat and fibrosis	Elongated and broad accumulation of fat cells about the implant site	Extensive fat completely surrounding the implant
**Irritancy score** = (Polymorphonuclear Cells + Lymphocytes + Plasma Cells + Macrophages + Giant Cells + Necrosis) × 2 + (Neovascularization + Fibrosis + Fatty Infiltrate)					

* *p* ≤ 0.05.

**Table 11:** Irritancy/Reactivity Grade.

Overall Irritancy Score	Irritancy/Reactivity Status
0.0 to 2.9	Minimal or no reaction (non-irritant)
3.0 to 8.9	Slight reaction (slight irritant)
9.0 to 15.0	Moderate reaction (moderate irritant)
>15.1	Severe reaction (severe irritant)

## Data Availability

The data presented in this study are available on request from the corresponding author.

## Abbreviations

BSM	Bone substitute material
β-TCP	beta-tricalcium phosphate
HY	Hyaluronic acid / hyaluronate
HMWHY	High molecular weight HY
HYAL	Hyaluronidase
GBR	Guided bone regeneration
MNGC	Multinucleated giant cell
VEGF	Vascular endothelial growth factor

## References

[1] Sakkas A., Wilde F., Heufelder M., Winter K., Schramm A. (2017). Autogenous bone grafts in oral implantology-is it still a “gold standard”? A consecutive review of 279 patients with 456 clinical procedures. Int. J. Implant Dent..

[2] Moore W.R., Graves S.E., Bain G.I. (2001). Synthetic bone graft substitutes. ANZ J. Surg..

[3] Miyamoto I., Funaki K., Yamauchi K., Kodama T., Takahashi T. (2012). Alveolar ridge reconstruction with titanium mesh and autogenous particulate bone graft: Computed tomography-based evaluations of augmented bone quality and quantity. Clin. Implant Dent. Relat. Res..

[4] Schaaf H., Lendeckel S., Howaldt H.P., Streckbein P. (2010). Donor site morbidity after bone harvesting from the anterior iliac crest. Oral Surg. Oral Med. Oral Pathol. Oral Radiol. Endod..

[5] Dimitriou R., Mataliotakis G.I., Angoules A.G., Kanakaris N.K., Giannoudis P.V. (2011). Complications following autologous bone graft harvesting from the iliac crest and using the RIA: A systematic review. Injury.

[6] Moussa N.T., Dym H. (2020). Maxillofacial Bone Grafting Materials. Dent. Clin. N. Am..

[7] Parikh S. (2002). Bone graft substitutes: Past, present, future. J. Postgrad. Med..

[8] Sukumar S., Drízhal I. (2008). Bone grafts in periodontal therapy. Acta Med..

[9] Lee J.H., Yi G.S., Lee J.W., Kim D.J. (2017). Physicochemical characterization of porcine bone-derived grafting material and comparison with bovine xenografts for dental applications. J. Periodontal. Implant Sci..

[10] Tadic D., Epple M. (2004). A thorough physicochemical characterisation of 14 calcium phosphate-based bone substitution materials in comparison to natural bone. Biomaterials.

[11] Barbeck M., Perić-Kačarević Ž., Kavehei F., Rider P., Najman S., Stojanović S., Rimashevskiy D., Wenisch S., Schnettler R. (2019). The effect of temperature treatment of xenogeneic bone substitute on the tissue response—A mini review. Acta Med. Median..

[12] Barbeck M., Udeabor S., Lorenz J., Schlee M., Holthaus M.G., Raetscho N., Choukroun J., Sader R., Kirkpatrick C.J., Ghanaati S. (2015). High-Temperature Sintering of Xenogeneic Bone Substitutes Leads to Increased Multinucleated Giant Cell Formation: In Vivo and Preliminary Clinical Results. J. Oral. Implant..

[13] Tawil G., Barbeck M., Unger R., Tawil P., Witte F. (2018). Sinus Floor Elevation Using the Lateral Approach and Window Repositioning and a Xenogeneic Bone Substitute as a Grafting Material: A Histologic, Histomorphometric, and Radiographic Analysis. Int. J. Oral. Maxillofac. Implant..

[14] Klein M.O., Kämmerer P.W., Götz H., Duschner H., Wagner W. (2013). Long-term bony integration and resorption kinetics of a xenogeneic bone substitute after sinus floor augmentation: Histomorphometric analyses of human biopsy specimens. Int. J. Periodontics Restor. Dent..

[15] Dahlin C., Simion M., Hatano N. (2010). Long-term follow-up on soft and hard tissue levels following guided bone regeneration treatment in combination with a xenogeneic filling material: A 5-year prospective clinical study. Clin. Implant Dent. Relat. Res..

[16] Yamada M., Egusa H. (2018). Current bone substitutes for implant dentistry. J. Prosthodont. Res..

[17] Larsson S., Hannink G. (2011). Injectable bone-graft substitutes: Current products, their characteristics and indications, and new developments. Injury.

[18] Kolk A., Handschel J., Drescher W., Rothamel D., Kloss F., Blessmann M., Heiland M., Wolff K.-D., Smeets R. (2012). Current trends and future perspectives of bone substitute materials–from space holders to innovative biomaterials. J. Cranio-Maxillofac. Surg..

[19] Low K.L., Tan S.H., Zein S.H.S., Roether J.A., Mourino V., Boccaccini A.R. (2010). Calcium phosphate-based composites as injectable bone substitute materials. J. Biomed. Mater. Res. Part B Appl. Biomater..

[20] Lorenz J., Barbeck M., Kirkpatrick C.J., Sader R., Lerner H., Ghanaati S. (2018). Injectable Bone Substitute Material on the Basis of ß-TCP and Hyaluronan Achieves Complete Bone Regeneration While Undergoing Nearly Complete Degradation. Int. J. Oral Maxillofac. Implant..

[21] Weiss P., Gauthier O., Bouler J.-M., Grimandi G., Daculsi G. (1999). Injectable bone substitute using a hydrophilic polymer. Bone.

[22] Clough B.H., McCarley M.R., Krause U., Zeitouni S., Froese J.J., McNeill E.P., Chaput C.D., Sampson H.W., Gregory C.A. (2015). Bone regeneration with osteogenically enhanced mesenchymal stem cells and their extracellular matrix proteins. J. Bone Miner. Res..

[23] Hidalgo-Bastida L.A., Cartmell S.H. (2010). Mesenchymal stem cells, osteoblasts and extracellular matrix proteins: Enhancing cell adhesion and differentiation for bone tissue engineering. Tissue Eng. Part B Rev..

[24] Ghanaati S., Barbeck M., Hilbig U., Hoffmann C., Unger R.E., Sader R.A., Peters F., Kirkpatrick C.J. (2011). An injectable bone substitute composed of beta-tricalcium phosphate granules, methylcellulose and hyaluronic acid inhibits connective tissue influx into its implantation bed in vivo. Acta Biomater..

[25] Barbeck M., Jung O., Smeets R., Gosau M., Schnettler R., Rider P., Houshmand A., Korzinskas T. (2020). Implantation of an Injectable Bone Substitute Material Enables Integration Following the Principles of Guided Bone Regeneration. Vivo.

[26] Aslan M., Şimşek G., Dayi E. (2006). The effect of hyaluronic acid-supplemented bone graft in bone healing: Experimental study in rabbits. J. Biomater. Appl..

[27] de Brito Bezerra B., Mendes Brazão M.A., de Campos M.L.G., Casati M.Z., Sallum E.A., Sallum A.W. (2012). Association of hyaluronic acid with a collagen scaffold may improve bone healing in critical-size bone defects. Clin. Oral Implant. Res..

[28] Chang Y.-L., Lo Y.-J., Feng S.-W., Huang Y.-C., Tsai H.-Y., Lin C.-T., Fan K.-H., Huang H.-M. (2016). Bone healing improvements using hyaluronic acid and hydroxyapatite/beta-tricalcium phosphate in combination: An animal study. Biomed Res. Int..

[29] Franz S., Rammelt S., Scharnweber D., Simon J.C. (2011). Immune responses to implants–a review of the implications for the design of immunomodulatory biomaterials. Biomaterials.

[30] Gardner A.B., Lee S.K., Woods E.C., Acharya A.P. (2013). Biomaterials-based modulation of the immune system. Biomed Res. Int..

[31] Kim H., Cha J., Jang M., Kim P. (2019). Hyaluronic acid-based extracellular matrix triggers spontaneous M2-like polarity of monocyte/macrophage. Biomater. Sci..

[32] Sieger D., Korzinskas T., Jung O., Stojanovic S., Wenisch S., Smeets R., Gosau M., Schnettler R., Najman S., Barbeck M. (2019). The Addition of High Doses of Hyaluronic Acid to a Biphasic Bone Substitute Decreases the Proinflammatory Tissue Response. Int. J. Mol. Sci..

[33] Brandt K.D., Smith G.N., Simon L.S. (2000). Intraarticular injection of hyaluronan as treatment for knee osteoarthritis: What is the evidence?. Arthritis Rheum.

[34] Felson D.T., Anderson J.J. (2002). Hyaluronate sodium injections for osteoarthritis: Hope, hype, and hard truths. Arch. Intern. Med..

[35] Barbeck M., Motta A., Migliaresi C., Sader R., Kirkpatrick C.J., Ghanaati S. (2016). Heterogeneity of biomaterial-induced multinucleated giant cells: Possible importance for the regeneration process?. J. Biomed. Mater. Res. Part A.

[36] Barbeck M., Udeabor S., Lorenz J., Kubesch A., Choukroun J., Sader R., Kirkpatrick C., Ghanaati S. (2014). Induction of multinucleated giant cells in response to small sized bovine bone substitute (Bio-Oss™) results in an enhanced early implantation bed vascularization. Ann. Maxillofac. Surg..

[37] Barbeck M., Serra T., Booms P., Stojanovic S., Najman S., Engel E., Sader R., Kirkpatrick C.J., Navarro M., Ghanaati S. (2017). Analysis of the in vitro degradation and the in vivo tissue response to bi-layered 3D-printed scaffolds combining PLA and biphasic PLA/bioglass components–Guidance of the inflammatory response as basis for osteochondral regeneration. Bioact. Mater..

[38] Al-Namnam N., Jayash S.N. (2019). Recent advances in bone graft substitute for oral and maxillofacial applications: A review. Int. J. Biosci..

[39] Liu M., Zeng X., Ma C., Yi H., Ali Z., Mou X., Li S., Deng Y., He N. (2017). Injectable hydrogels for cartilage and bone tissue engineering. Bone Res..

[40] Misch C.E., Dietsh F. (1993). Bone-grafting materials in implant dentistry. Implant Dent..

[41] Sheikh Z., Najeeb S., Khurshid Z., Verma V., Rashid H., Glogauer M. (2015). Biodegradable materials for bone repair and tissue engineering applications. Materials.

[42] Bouler J.-M., Pilet P., Gauthier O., Verron E. (2017). Biphasic calcium phosphate ceramics for bone reconstruction: A review of biological response. Acta Biomater..

[43] Sheikh Z., Hamdan N., Ikeda Y., Grynpas M., Ganss B., Glogauer M. (2017). Natural graft tissues and synthetic biomaterials for periodontal and alveolar bone reconstructive applications: A review. Biomater. Res..

[44] Taschieri S., Del Fabbro M., Testori T., Weinstein R. (2007). Efficacy of xenogeneic bone grafting with guided tissue regeneration in the management of bone defects after surgical endodontics. J. Oral Maxillofac. Surg..

[45] Zhang N., Ma L., Liu X., Jiang X., Yu Z., Zhao D., Zhang L., Zhang C., Huang F. (2018). In vitro and in vivo evaluation of xenogeneic bone putty with the carrier of hydrogel derived from demineralized bone matrix. Cell Tissue Bank.

[46] Mondal S., Haridas N., Letha S.S., Vijith V., Rajmohan G., Rosemary M. (2016). Development of injectable high molecular weight hyaluronic acid hydrogels for cartilage regeneration. J. Macromol. Sci. Part A.

[47] Zhai P., Peng X., Li B., Liu Y., Sun H., Li X. (2020). The application of hyaluronic acid in bone regeneration. Int. J. Biol. Macromol..

[48] Pardue E.L., Ibrahim S., Ramamurthi A. (2008). Role of hyaluronan in angiogenesis and its utility to angiogenic tissue engineering. Organogenesis.

[49] Özan F., Şençimen M., Gülses A., Ayna M. (2016). Guided bone regeneration technique using hyaluronic acid in oral implantology. A Textb. Adv. Oral Maxillofac. Surg..

[50] Snetkov P., Zakharova K., Morozkina S., Olekhnovich R., Uspenskaya M. (2020). Hyaluronic Acid: The Influence of Molecular Weight on Structural, Physical, Physico-Chemical, and Degradable Properties of Biopolymer. Polymers.

[51] Stern R., Jedrzejas M.J. (2006). Hyaluronidases: Their genomics, structures, and mechanisms of action. Chem. Rev..

[52] Rothamel D., Schwarz F., Herten M., Berndsen K., Fienitz T., Ritter L., Dreiseidler T., Zöller J. Impact of Citric Acid Etching on Biocompatibility and Osseous Organisation of a Natural Bovine Bone Mineral: Preliminary Results of an In-Vitro/In-Vivo Study. Proceedings of the World Congress on Medical Physics and Biomedical Engineering.

[53] Barbeck M., Hoffmann C., Sader R., Peters F., Hubner W.D., Kirkpatrick C.J., Ghanaati S. (2016). Injectable Bone Substitute Based on beta-TCP Combined With a Hyaluronan-Containing Hydrogel Contributes to Regeneration of a Critical Bone Size Defect Towards Restitutio ad Integrum. J. Oral Implant..

[54] Sarkar K., Xue Y., Sant S. (2017). Host response to synthetic versus natural biomaterials. The Immune Response to Implanted Materials and Devices.

[55] Ghanaati S., Barbeck M., Booms P., Lorenz J., Kirkpatrick C.J., Sader R.A. (2014). Potential lack of “standardized” processing techniques for production of allogeneic and xenogeneic bone blocks for application in humans. Acta Biomater..

[56] Shao A., Ling Y., Xu L., Liu S., Fan C., Wang Z., Xu B., Wang C. (2018). Xenogeneic bone matrix immune risk assessment using GGTA1 knockout mice. Artif Cells Nanomed Biotechnol.

[57] Barbeck M., Batinic M., Alkildani S., Pantermehl S., Jung O. (2021). Analysis of the systemic and local immune response to different bone substitute classes.

[58] Perić Kačarević Ž., Rider P., Alkildani S., Retnasingh S., Pejakić M., Schnettler R., Gosau M., Smeets R., Jung O., Barbeck M. (2020). An introduction to bone tissue engineering. Int. J. Artif. Organs.

[59] Ghanaati S., Barbeck M., Orth C., Willershausen I., Thimm B.W., Hoffmann C., Rasic A., Sader R.A., Unger R.E., Peters F. (2010). Influence of beta-tricalcium phosphate granule size and morphology on tissue reaction in vivo. Acta Biomater..

[60] Ghanaati S., Barbeck M., Detsch R., Deisinger U., Hilbig U., Rausch V., Sader R., Unger R.E., Ziegler G., Kirkpatrick C.J. (2012). The chemical composition of synthetic bone substitutes influences tissue reactions in vivo: Histological and histomorphometrical analysis of the cellular inflammatory response to hydroxyapatite, beta-tricalcium phosphate and biphasic calcium phosphate ceramics. Biomed. Mater..

[61] Barbeck M., Dard M., Kokkinopoulou M., Markl J., Booms P., Sader R.A., Kirkpatrick C.J., Ghanaati S. (2015). Small-sized granules of biphasic bone substitutes support fast implant bed vascularization. Biomatter.

[62] Rosengren A., Danielsen N., Bjursten L.M. (1997). Inflammatory reaction dependence on implant localization in rat soft tissue models. Biomaterials.

[63] Kapogianni E., Barbeck M., Jung O., Arslan A., Kuhnel L., Xiong X., Krastev R., Friedrich R.E., Schnettler R., Fienitz T. (2019). Comparison of Material-mediated Bone Regeneration Capacities of Sintered and Non-sintered Xenogeneic Bone Substitutes via 2D and 3D Data. Vivo.

[64] Lindner C., PrOhl A., Abels M., Löffler T., Batinic M., Jung O., Barbeck M. (2020). Specialized Histological and Histomorphometrical Analytical Methods for Biocompatibility Testing of Biomaterials for Maxillofacial Surgery in (Pre-) Clinical Studies. Vivo.

